# Supplementary material for: MCT6 is an intestinal Lac-Phe exporter required for metformin-associated weight loss
Source: bioRxiv. 2026 Sep 28:2026.09.22.753556. Preprint. [Version 1] doi: 10.64898/2026.09.22.753556 (PMC13648347; doi:10.64898/2026.09.22.753556)

bioRxiv preprint doi: https://doi.org/10.64898/2026.09.22.753556; this version posted September 28, 2026. The copyright holder for this preprint (which was not certified by peer review) is the author/funder, who has granted bioRxiv a license to display the preprint in perpetuity. It is made available under a CC-BY 4.0 International license.

Supplementary Figure Legends

Figure S1. Additional characterization of MCT6 overexpressing or knockout cells.

(A,B) Levels of Lac-Phe in conditioned medium (A) or cell lysates (B) of transfected HEK293T cells expressing mCherry (Control), SLC16A5, or SLC17A3. N = 4/group. (C,D) Levels of lactate in conditioned medium (C) or cell lysates (D) of transfected HEK293T cells expressing mCherry (Control), SLC16A5, or SLC17A3. N = 4/group. (E,F) Levels of phenylalanine in conditioned medium (E) or cell lysates (F) of transfected HEK293T cells expressing mCherry (Control), SLC16A5, or SLC17A3. N = 4/group. (G) Anti-CNDP2 (top) and anti-tubulin (bottom) blotting of HEK293T cells transfected with SLC16A5 or mCherry (Control). (H) Phylogenetic tree of the SLC16/MCT transporter family. The phylogeny was constructed using the Maximum Likelihood method using protein sequences. (I) Expression levels of *Actb*, *Gapdh*, and *Slc16a5* mRNA in HEK293T (I) and HCT116 (J) cells. N = 3 technical replicates. (K) Anti-CNDP2 (top) and anti-tubulin (bottom) blotting of HEK293T and HCT116 cells with or without doxycycline-inducible MCT6 expression. (L,M) Lac-Phe synthesis activity of cell lysates of HEK293T (L) and HCT116 (M) cells with or without doxycycline-inducible MCT6 expression. N = 3/group. (N) Indel rate of HCT116 KO1 and KO2 cells. (O) Anti-CNDP2 (top) and anti-tubulin (bottom) blotting of HCT116 cells with corresponding genotypes. (P) Lac-Phe synthesis activity of cell lysates of HCT116 cells with corresponding genotypes. N = 3/group. Data are shown as mean ± SEM. ** $p < 0.01$, **** $p < 0.0001$. In (A-F,P) *p*-values were calculated from one-way ANOVA with post hoc Dunnett's multiple comparisons test. In (L,M), *p*-values were calculated with two-tailed unpaired t tests.

Figure S2. Additional characterization of MCT6-mediated Lac-Phe transport.

(A) D_3-Lac-Phe transport activity in MCT6-KO cells (black line) or in KO cells reconstituted with MCT6 (pink line) with different D_3-Lac-Phe concentrations for 10 minute incubation (A) or with

bioRxiv preprint doi: https://doi.org/10.64898/2026.09.22.753556; this version posted September 28, 2026. The copyright holder for this preprint (which was not certified by peer review) is the author/funder, who has granted bioRxiv a license to display the preprint in perpetuity. It is made available under a CC-BY 4.0 International license.

different incubation times and 1 mM D_3-Lac-Phe (B). MCT6-specific transport activity is shown in the teal line. N = 3/time point. (C) MCT6-specific D_3-Lac-Phe transport activity in a mammalian cell-based transport assay with 1 mM D_3-Lac-Phe in the presence of various concentrations of bumetanide for 10 minute incubation. N = 3/group. (D) MCT6-specific D_3-Lac-Phe transport activity in a mammalian cell-based transport assay with 1 mM D_3-Lac-Phe with standard HBSS (Control), Na^+-free HBSS (NMDG), Cl^--free HBSS (Gluconate), or Na^+,Cl^--free HBSS (Mannitol). N = 3/group. (E) MCT6-specific Lac-Phe transport activity in a mammalian cell-based transport assay with 1 mM Lac-Phe at different pHs for 10 minutes. N = 3/group. (F) MCT6-specific D_3-Lac-Phe transport activity in a mammalian cell-based transport assay with 1 mM D_3-Lac-Phe at pH 6.0 or 7.4. Valinomycin and nigericin in a high KCl buffer was used to dissipate both the membrane potential and proton gradient. N = 3/group. (G) Inward-open conformation of mMCT6 based on an AlphaFold-predicted structure. (H) Additional top-ranked molecular docking poses of mMCT6 and Lac-Phe. Individual residues (orange) within 4Å of Lac-Phe (blue) are highlighted. (I) MCT6-specific bumetanide transport activity of WT, G347D, R288W, and G347W MCT6 mutants. N = 3/group. (J) Physiochemical properties of putative substrate-binding cavities in MCT6, SLC17A1, SLC17A3, and ABCC5 using SiteMap. SiteScore, overall composite site quality score; Size, number of site points used to define the pocket; Dscore, druggability score; Volume, physical volume of the identified pocket; Exposure, ratio of the pocket exposed to solvent; Enclosure, ratio of the pocket surrounded by protein structure; Contact, degree of direct van der Waals contact; Phobic, hydrophobic character score of the pocket lining; Philic, hydrophilic character score of the pocket lining; Balance, ratio of Phobic to Philic; Don/Acc, ratio of hydrogen-bond donor character to acceptor character. For (A-F) and (I), MCT6-specific transport activity was determined by subtraction of the background activity in the MCT6-KO cells. Data are shown as mean ± SEM. *** $p < 0.001$, **** $p < 0.0001$. In (D), p-values were calculated from one-way ANOVA with post hoc Dunnett's multiple comparisons test, compared to Control. In (E,F), p-values were calculated from one-way

bioRxiv preprint doi: https://doi.org/10.64898/2026.09.22.753556; this version posted September 28, 2026. The copyright holder for this preprint (which was not certified by peer review) is the author/funder, who has granted bioRxiv a license to display the preprint in perpetuity. It is made available under a CC-BY 4.0 International license.

ANOVA with post hoc Tukey's multiple comparisons test, different letters represent *p*-values < 0.05. In (I), *p*-values were calculated from mixed-effects analysis with Tukey's multiple comparisons tests.

Figure S3. Additional molecular characterization of MCT6-KO mice.

(A) Plasma metformin levels in WT and MCT6-KO mice after a single oral administration (300 mg/kg, p.o.) (B-E) Levels of Lac-Phe in the gut (B), muscle (C), liver (D), and brain (E) at both basal and metformin-induced (30 min after a single administration of metformin, 300 mg/kg, p.o.) states in 14-20 week-old female WT and MCT6-KO mice. (F,G) Cumulative change in body weight (F) and food intake (G) in 4-6 week old male WT and MCT6-KO mice on chow diet. N = 12 for WT and N = 7 for KO. (H,I) Cumulative change in body weight (H) and food intake (I) in 4-6 week old male WT and MCT6-KO mice on high fat diet (HFD). N = 6 for WT and N = 10 for KO. (J-N) 24 hour locomotor activity (J), oxygen consumption (K), carbon dioxide production (L), respiratory exchange ratio (RER, M), and food intake (N) of 25-30 week-old male WT or MCT6-KO mice after 12-16 weeks of high fat diet. N = 7-8/group. (O-R) Relative mRNA expression of indicated genes in liver (O,Q), quadriceps muscle (P) or epididymal white adipose tissue (R) from 25-30 week-old male WT and MCT6-KO mice on HFD for 12-16 weeks. N = 5/group. (S-X) Levels of AST (S), ALT (T), triglycerides (U), HDL (V), LDL (W), cholesterol (X) from 25-30 week old male WT and MCT6-KO mice on HFD for 12-16 weeks. N = 5/group. (Y,Z) Cumulative change in body weight (Y) and food intake (Z) in 20-24 week-old male WT and MCT6-KO lean mice following chronic metformin treatment (300 mg/kg daily, p.o.). N = 6/group. Starting body weights were WT: 31.5 ± 1.9 g, KO: 31.6 ± 1.4 g (mean ± SEM). Data are shown as mean ± SEM. ** $p < 0.01$. In (B-E,O-R), *p*-values were calculated with two-way ANOVA with post hoc Sidak's multiple comparisons test. In (F-I,Y,Z), *p*-values were calculated from two-way ANOVA and reporting the effect of genotype. In (J-N,S-X), *p*-values were calculated from two-tailed unpaired t-test.

bioRxiv preprint doi: https://doi.org/10.64898/2026.09.22.753556; this version posted September 28, 2026. The copyright holder for this preprint (which was not certified by peer review) is the author/funder, who has granted bioRxiv a license to display the preprint in perpetuity. It is made available under a CC-BY 4.0 International license.

Figure S4. Characterization of additional MCT6 substrates.

(A) MS/MS fragmentation of the endogenous *m/z* = 252.09 (left, top) and authentic Lac-Tyr standard (left, bottom) and co-elution of the endogenous peak and synthesized standard (right). (B) MS/MS fragmentation of the endogenous *m/z* = 202.11 (left and middle, top), authentic Lac-Leu (left, bottom) and Lac-Ile (middle, bottom) standard, and co-elution of the endogenous peak and synthesized standards (right). (C) MS/MS fragmentation of the endogenous *m/z* = 188.09 (left, top) and authentic Lac-Val standard (left, bottom) and co-elution of the endogenous peak and synthesized standard (right). (D) MS/MS fragmentation of the endogenous *m/z* = 275.10 (left, top) and authentic Lac-Trp standard (left, bottom) and co-elution of the endogenous peak and synthesized standard (right). (E) MS/MS fragmentation of the endogenous *m/z* = 190.05 (left, top) and authentic Ac-Met standard (left, bottom) and co-elution of the endogenous peak and synthesized standard (right). (F) MS/MS fragmentation of the endogenous *m/z* = 181.05 (left, top) and authentic HPLA standard (left, bottom) and co-elution of the endogenous peak and synthesized standard (right). (G-M) Transport activity of *X. laevis* oocytes with different concentrations of additional MCT6 substrates: Lac-Tyr (G), Lac-Trp (H), Lac-Leu (I), Lac-Ile (J), Lac-Val (K), , Ac-Met (L), HPLA (M). N = 3-5/group (N-T) Transport activity with a mammalian cell-based transport assay with different concentrations of additional MCT6 substrates: Lac-Tyr (N), Lac-Trp (O), Lac-Leu (P), Lac-Ile (Q), Lac-Val (R), Ac-Met (S), HPLA (T). For (G-T), MCT6-specific transport activity was determined by subtraction of the background activity in water-injected oocytes (G-M) or MCT6-KO cells (N-T). N = 3/group. Data are shown as mean ± SEM.

Figure S5. Biochemical characterization of MCT6-KO mice.

(A-D) Levels of MCT6 substrates in the gut (A), muscle (B), liver (C), and brain (D) of 14-20 week-old female WT and MCT6-KO mice under basal state or 30 min after a single administration of metformin (300 mg/kg, p.o.). Levels were normalized to WT mice under basal

bioRxiv preprint doi: https://doi.org/10.64898/2026.09.22.753556; this version posted September 28, 2026. The copyright holder for this preprint (which was not certified by peer review) is the author/funder, who has granted bioRxiv a license to display the preprint in perpetuity. It is made available under a CC-BY 4.0 International license.

state, with $\log_2$ transformation. Lac-Trp was below detection limit in all four tissues, Lac-Tyr and Lac-Val were below detection limit in brain tissues. (E-I) Levels of metabolites in the blood plasma (E), gut (F), muscle (G), liver (H), and brain (I) of 14-20 week-old female WT and MCT6-KO mice under basal state or 30 min after a single administration of metformin (300 mg/kg, p.o.). Levels were normalized to WT mice under basal state, with $\log_2$ transformation. Glc, glucose; Lac, lactate; Suc, succinate; UA, uric acid; Orn, ornithine; Cit, citrulline; Tau, taurine; Ala, alanine; Asp, aspartic acid; Gly, glycine; Gln, glutamine; Glu, glutamic acid; Ile, isoleucine; Leu, leucine; Phe, phenylalanine; Ser, serine; Trp, tryptophan; Tyr, tyrosine; Val, valine; C16:0, palmitate; C18:0, stearate; C18:1, oleate; C20:4, arachidonate. Data are shown as mean ± SEM. * $p < 0.05$. In all panels, p-values were calculated separately for Basal state and Metformin state, from two-way ANOVA with post hoc Šídák's multiple comparisons test.

Figure S6. Effect of treadmill exercise in MCT6-KO mice.

(A) Schematic of speed and incline in the chronic/moderate intensity running protocol for mice. (B,C) Cumulative body weight change (B) and cumulative food intake (C) in 10-14 week old male WT and MCT6-KO mice following a combined chronic treadmill running and high fat diet feeding protocol. N = 8 for WT and N = 7 for KO. (D-J) Plasma levels of Lac-Phe and additional MCT6 substrates at the indicated time point from 8-12 week old male WT and MCT6-KO mice before (time 0) and after a single bout of moderate intensity treadmill running for 30 min. N = 8 for WT, N = 6 for KO. (K-N) Levels of MCT6 substrates in the gut (K), muscle (L), liver (M), and brain (N) of 14-20 week-old female WT and MCT6-KO mice under basal state or after a single bout of treadmill running for 30 min. Levels were normalized to WT mice under basal state, with $\log_2$ transformation. N = 6 for WT, N = 7 for KO. (O-S) Levels of metabolites in the blood plasma (O), gut (P), muscle (Q), liver (R), and brain (S) of 14-20 week-old female WT and MCT6-KO mice under basal state or after a single bout of treadmill running for 30 min. Levels were normalized to WT mice under basal state, with $\log_2$ transformation. N = 6 for WT, N = 7 for KO.

bioRxiv preprint doi: https://doi.org/10.64898/2026.09.22.753556; this version posted September 28, 2026. The copyright holder for this preprint (which was not certified by peer review) is the author/funder, who has granted bioRxiv a license to display the preprint in perpetuity. It is made available under a CC-BY 4.0 International license.

Glc, glucose; Lac, lactate; Suc, succinate; UA, uric acid; Orn, ornithine; Cit, citrulline; Tau, taurine; Ala, alanine; Asp, aspartic acid; Gly, glycine; Gln, glutamine; Glu, glutamic acid; Ile, isoleucine; Leu, leucine; Phe, phenylalanine; Ser, serine; Trp, tryptophan; Tyr, tyrosine; Val, valine; C16:0, palmitate; C18:0, stearate; C18:1, oleate; C20:4, arachidonate. (T) Schematic of speed and incline in the acute/maximum intensity running protocol for mice. (U-AA) Plasma levels of Lac-Phe and additional MCT6 substrates at the indicated time point from 11-13 week old male WT and MCT6-KO mice before (time 0) and after a single bout of acute/maximum intensity treadmill running. N = 8 for WT, N = 6 for KO. Running time was 29.65 ± 1.6 min for WT, and 29.76 ± 1.7 min for KO. (AB,AC) Change of *Slc16a5* mRNA levels in the gut (AB) or muscle (AC) 30 min after a single administration of metformin (300 mg/kg, p.o.), or a single bout of moderate intensity treadmill running for 30 min. N = 5-6/group. Data are shown as mean ± SEM. ** $p < 0.01$. In (B-J) and (U-AA), *p*-values were calculated from two-way ANOVA and reporting the effect of genotype. In (K-S), *p*-values were calculated from two-way ANOVA with post hoc Šídák's multiple comparisons test. In (AB,AC), *p*-values were calculated from one-way ANOVA with Holm-Šídák's multiple comparisons test.

Figure S7. Additional characterization of intestine epithelial-specific MCT6-KO mice.

(A) Relative *Slc16a5/Mct6* gene expression (mRNA) level in mouse tissues based on Proteomicsdb dataset. (B) *Slc16a5/Mct6* gene and *Cndp2* gene mRNA gene expression level in single cell mouse small intestine dataset (Haber et al. 2017). (C-H) Plasma levels of additional MCT6 substrates at the indicated time points from 7-8 week-old male $Vil1^{Cre+/-}$; $Slc16a5^{fl/fl}$ mice and $Slc16a5^{fl/fl}$ controls before (time 0) and after metformin administration (300mg/kg, p.o.). N = 5-6/group. Data are shown as mean ± SEM. In (C-H), *p*-values were calculated from two-way ANOVA and reporting the effect of genotype.

bioRxiv preprint doi: https://doi.org/10.64898/2026.09.22.753556; this version posted September 28, 2026. The copyright holder for this preprint (which was not certified by peer review) is the author/funder, who has granted bioRxiv a license to display the preprint in perpetuity. It is made available under a CC-BY 4.0 International license.

Table S1. *Cndp2* and transporter co-expression analysis in the gut, related to Fig. 1A.

Table S2. Untargeted profiling of MCT6 substrates, related to Fig. 5A.

bioRxiv preprint doi: https://doi.org/10.64898/2026.09.22.753556; this version posted September 28, 2026. The copyright holder for this preprint (which was not certified by peer review) is the author/funder, who has granted bioRxiv a license to display the preprint in perpetuity. It is made available under a CC-BY 4.0 International license.

Figure S1

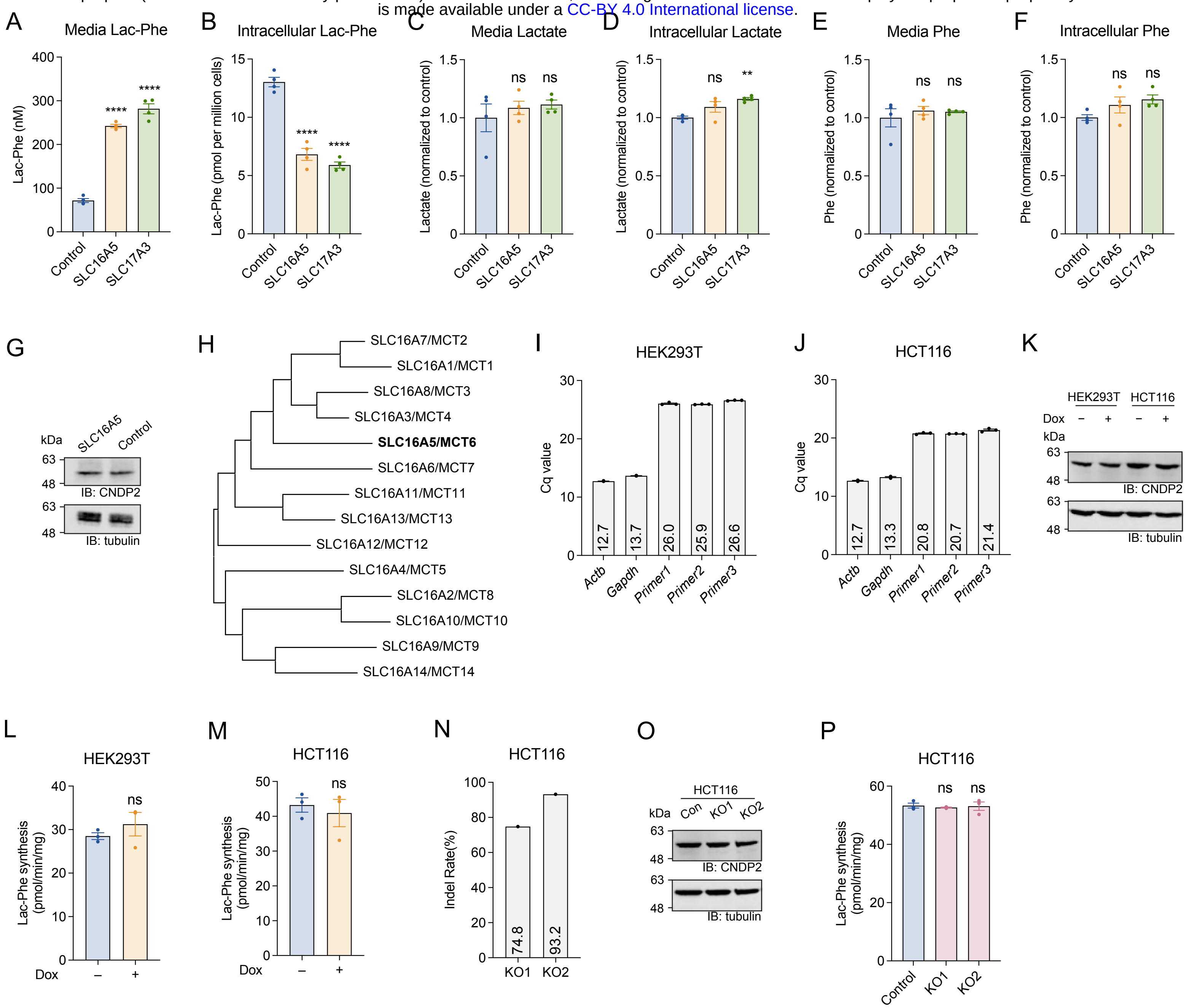

bioRxiv preprint doi: https://doi.org/10.64898/2026.09.22.753556; this version posted September 28, 2026. The copyright holder for this preprint (which was not certified by peer review) is the author/funder, who has granted bioRxiv a license to display the preprint in perpetuity. It is made available under a CC-BY 4.0 International license.

Figure S2

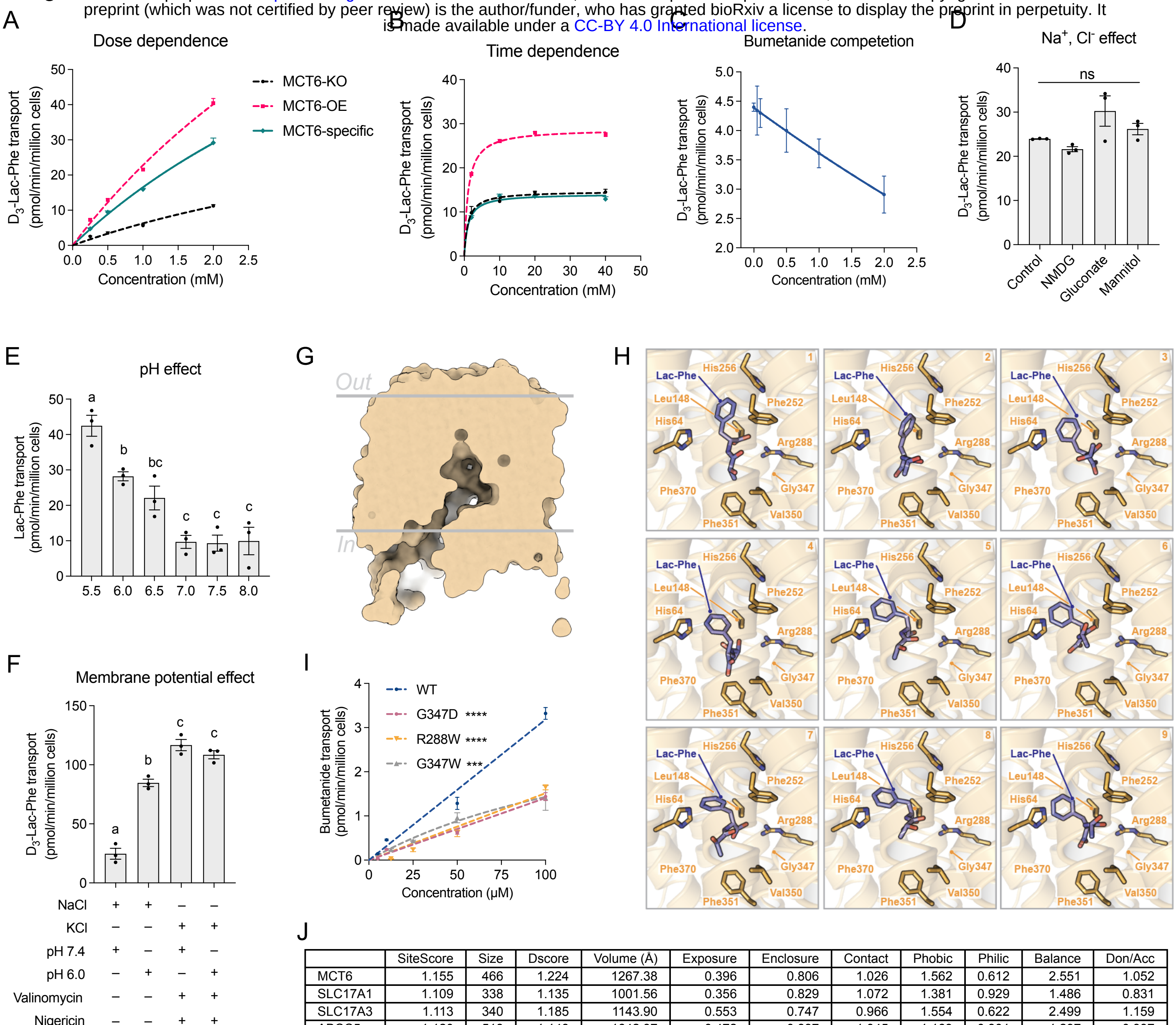

J

	SiteScore	Size	Dscore	Volume (Å)	Exposure	Enclosure	Contact	Phobic	Philic	Balance	Don/Acc
MCT6	1.155	466	1.224	1267.38	0.396	0.806	1.026	1.562	0.612	2.551	1.052
SLC17A1	1.109	338	1.135	1001.56	0.356	0.829	1.072	1.381	0.929	1.486	0.831
SLC17A3	1.113	340	1.185	1143.90	0.553	0.747	0.966	1.554	0.622	2.499	1.159
ABCC5	1.120	518	1.148	1642.97	0.472	0.837	1.045	1.169	0.901	1.297	0.997

Figure S3

bioRxiv preprint doi: https://doi.org/10.64898/2026.09.22.753556; this version posted September 28, 2026. The copyright holder for this preprint (which was not certified by peer review) is the author/funder, who has granted bioRxiv a license to display the preprint in perpetuity. It is made available under a CC-BY 4.0 International license.

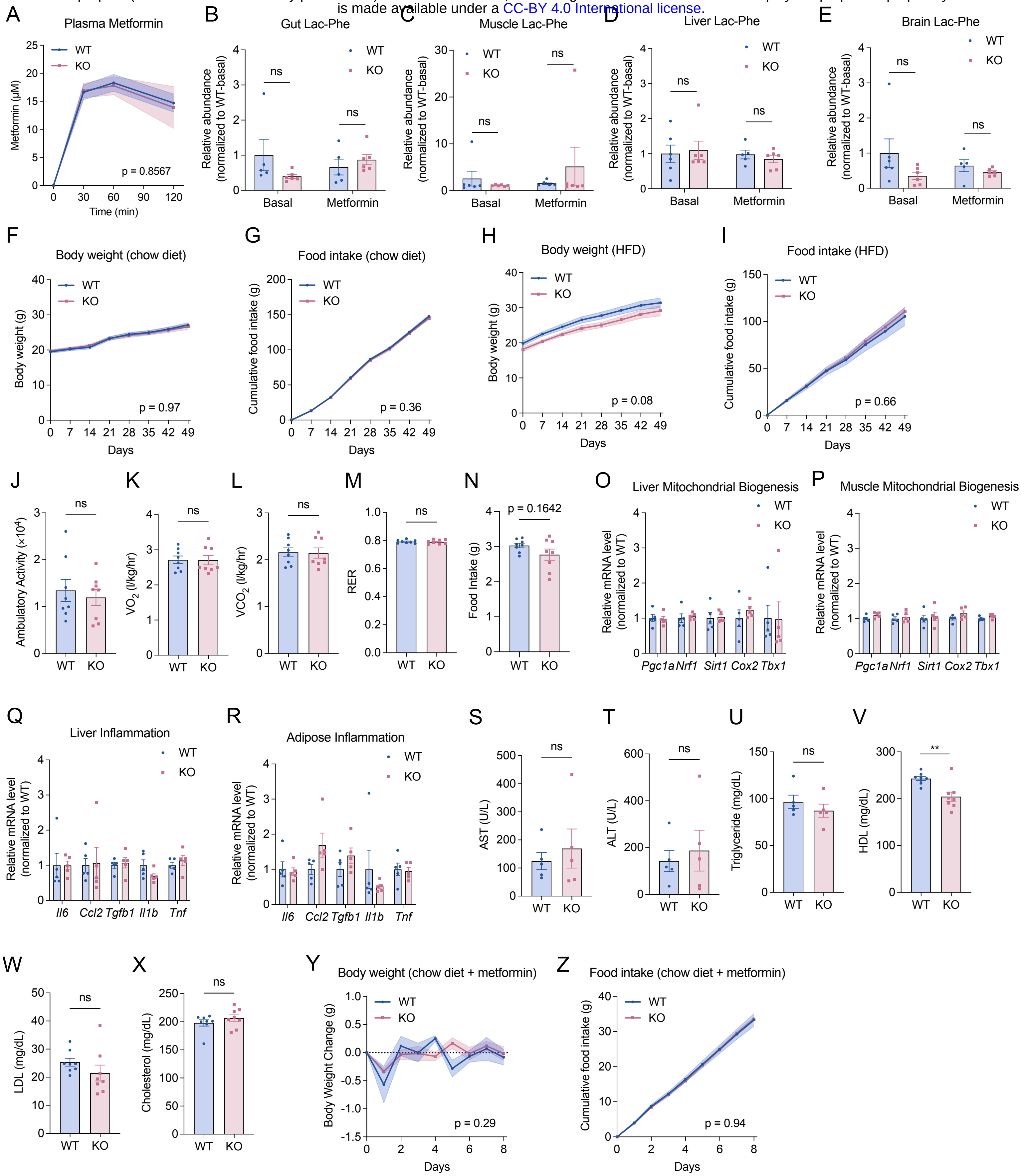

bioRxiv preprint doi: https://doi.org/10.64898/2026.09.22.753556; this version posted September 28, 2026. The copyright holder for this preprint (which was not certified by peer review) is the author/funder, who has granted bioRxiv a license to display the preprint in perpetuity. It is made available under a CC-BY 4.0 International license.

Figure S4

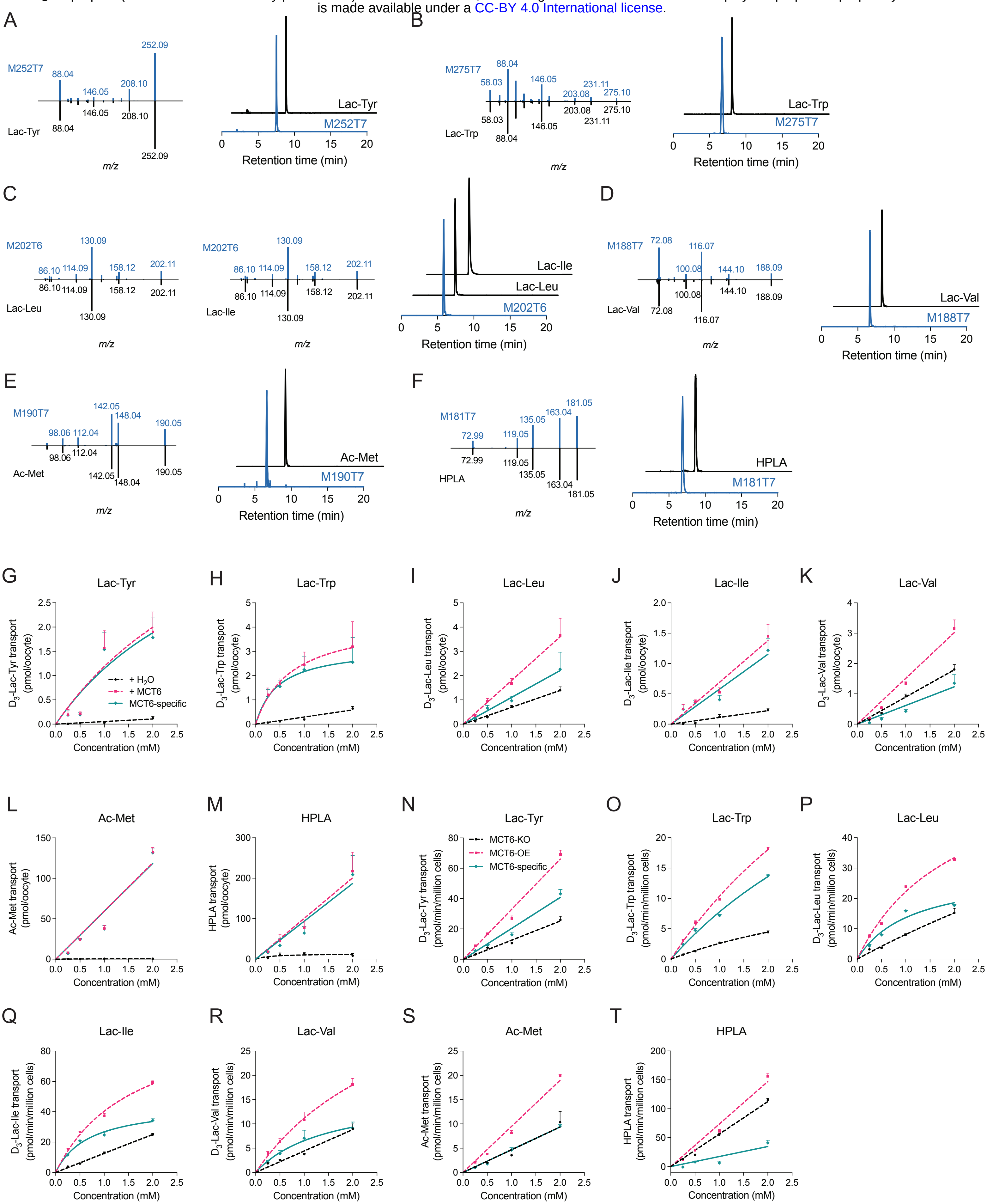

bioRxiv preprint doi: https://doi.org/10.64898/2026.09.22.753556; this version posted September 28, 2026. The copyright holder for this preprint (which was not certified by peer review) is the author/funder, who has granted bioRxiv a license to display the preprint in perpetuity. It is made available under a CC-BY 4.0 International license.

Figure S5

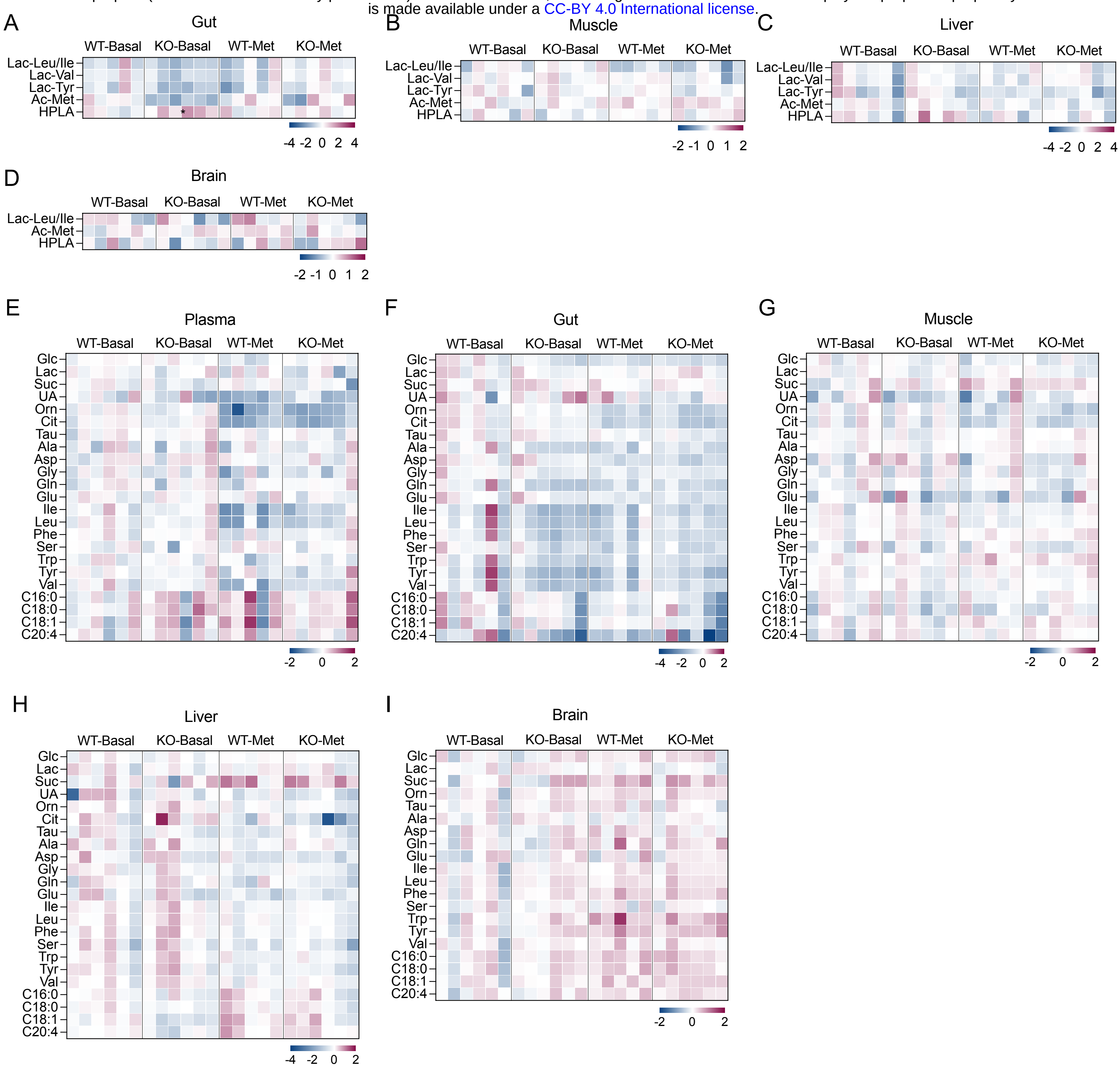

bioRxiv preprint doi: https://doi.org/10.64898/2026.09.22.753556; this version posted September 28, 2026. The copyright holder for this preprint (which was not certified by peer review) is the author/funder, who has granted bioRxiv a license to display the preprint in perpetuity. It is made available under a CC-BY 4.0 International license.

Figure S6

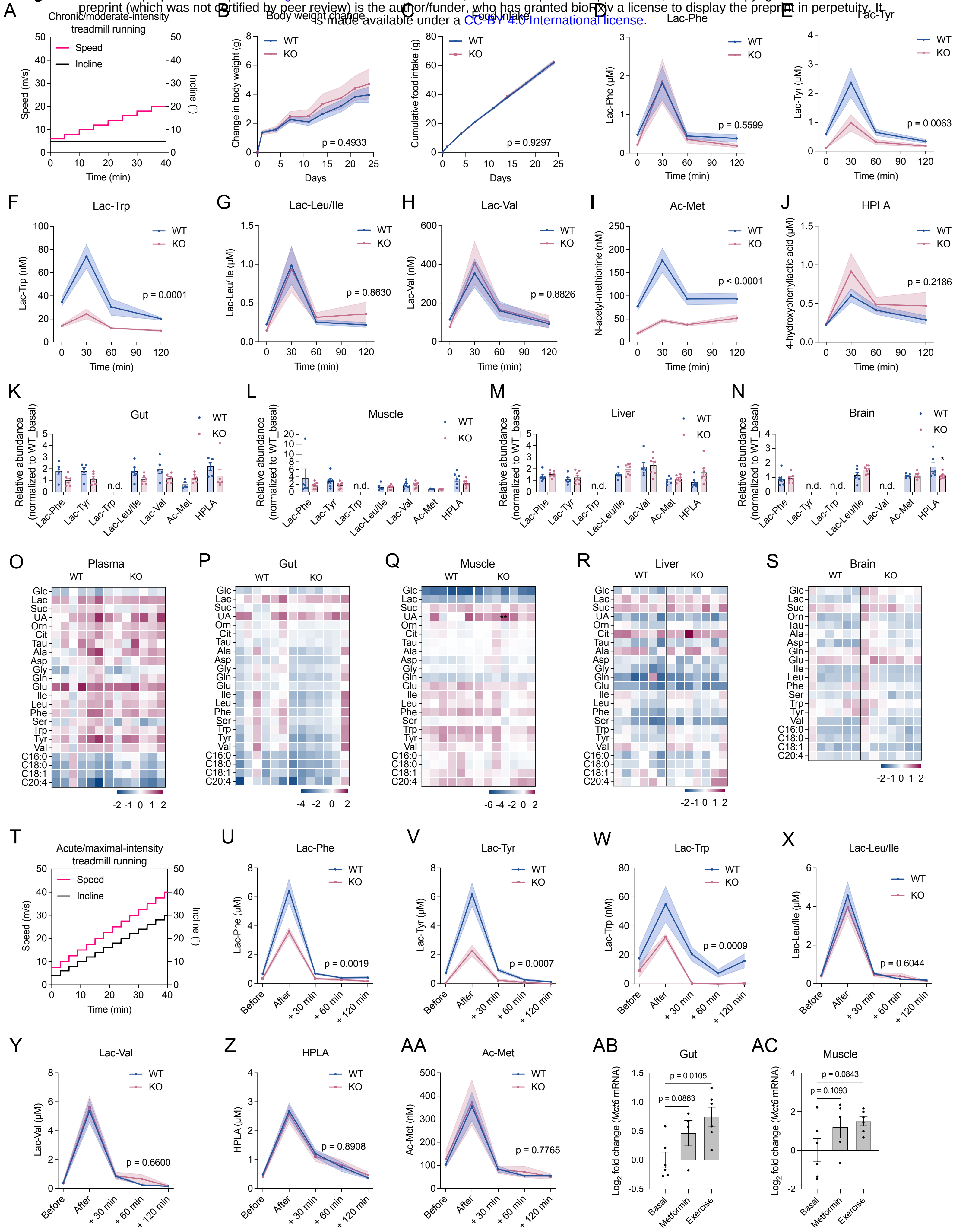

bioRxiv preprint doi: https://doi.org/10.64898/2026.09.22.753556; this version posted September 28, 2026. The copyright holder for this preprint (which was not certified by peer review) is the author/funder, who has granted bioRxiv a license to display the preprint in perpetuity. It is made available under a CC-BY 4.0 International license.

Figure S7

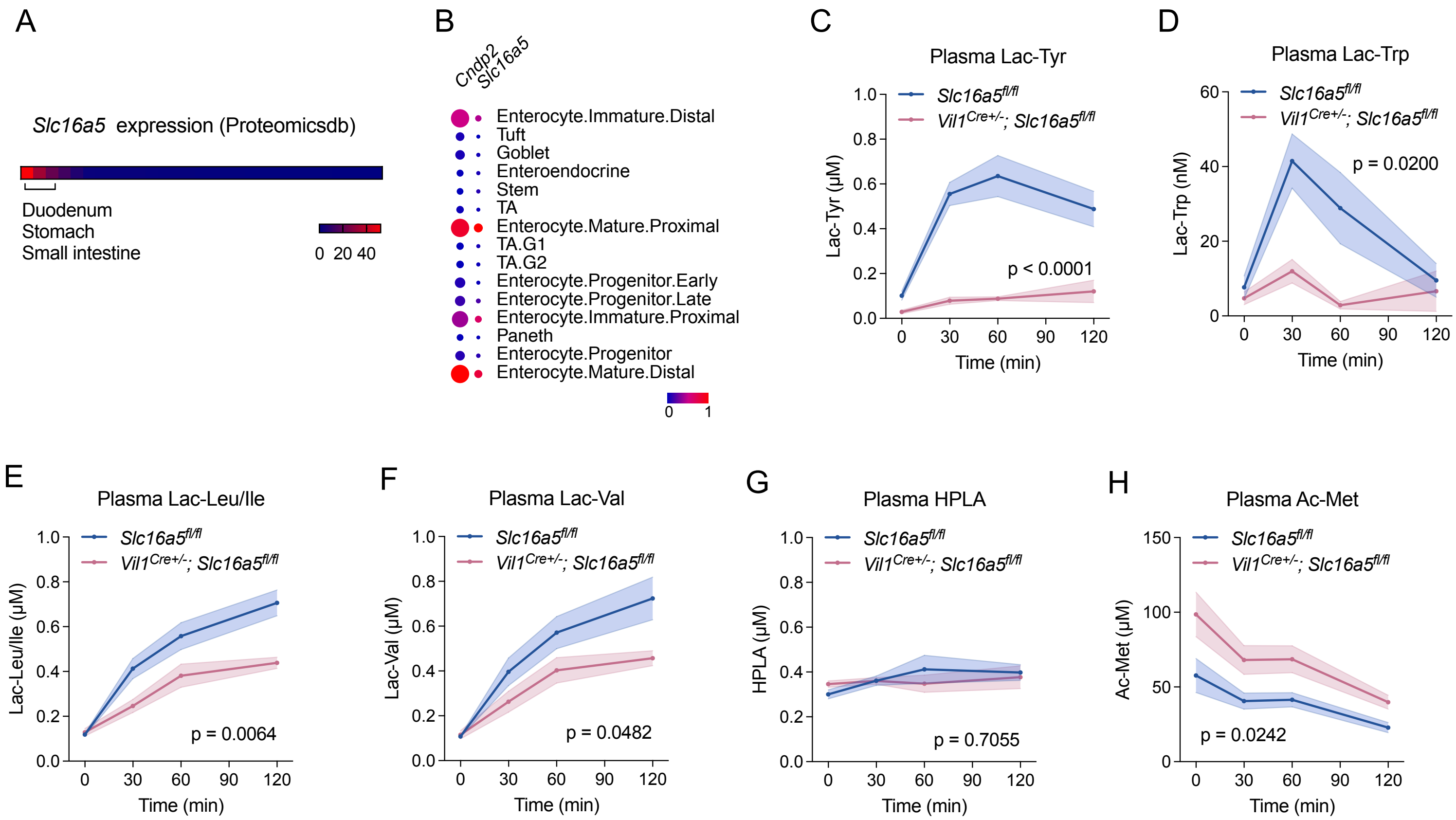

Supplement: Supplementary file 1 [file NIHPP2026.09.22.753556v1-supplement-3.pdf]
